# Silk-Fibroin-Based Strategies for Myocardial Infarction Repair: A Comprehensive Review

**DOI:** 10.3390/ijms27062885

**Published:** 2026-03-23

**Authors:** Shuyan Piao, Yanan Gao

**Affiliations:** 1 College of Basic Medical Sciences, Jilin University, Changchun 130021, China; piao2005@icloud.com; 2 Engineering Research Center of Tropical Medicine Innovation and Transformation of Ministry of Education & Hainan Provincial Key Laboratory of Research and Development on Tropical Herbs, School of Pharmacy, Hainan Medical University, Haikou 571199, China

**Keywords:** silk fibroin, myocardial infarction, cardiac tissue engineering, patch, hydrogel

## Abstract

Myocardial infarction is a major cardiovascular event that leads to heart failure and death. Although current vascular regeneration and pharmacological therapies can salvage some myocardial tissue, they cannot effectively reverse established necrosis, fibrosis, or adverse ventricular remodeling, thus necessitating novel repair strategies. Silk fibroin (SF), a natural biomaterial, has emerged as an ideal substrate for cardiac tissue engineering owing to its excellent biocompatibility, tunable mechanical properties, and controllable biodegradability. This paper systematically reviews SF-based myocardial repair strategies: SF cardiac patches can be directly applied to infarct areas, providing mechanical support and delivering bioactive substances, while injectable SF hydrogels can be formed in situ via minimally invasive methods, serving as three-dimensional delivery vehicles for cells or drugs. These approaches synergistically promote cardiac repair through multiple mechanisms, including active regulation of inflammation, promotion of angiogenesis, and inhibition of fibrosis. Future development of SF-based therapies will focus on creating smart responsive materials, constructing biomimetic structures via advanced biomanufacturing techniques, and accelerating clinical translation, thereby providing comprehensive solutions for myocardial infarction repair.

## 1. Introduction

### 1.1. Background of Myocardial Infarction

Myocardial infarction (MI) represents the most severe form of cardiovascular disease globally, causing approximately 9 million deaths annually, with its disease burden markedly concentrated in low- and middle-income countries. The pathological core of this condition lies in the interruption of myocardial blood flow triggered by coronary plaque rupture, leading to irreversible myocardial cell necrosis [1]. This initial injury triggers a vicious cycle involving intense inflammation, oxidative stress, apoptosis, and abnormal fibrosis. Ultimately, it leads to ventricular remodeling, characterized by ventricular dilation, wall thinning, and geometric distortion, which can progress to chronic heart failure [2]. In China, coronary heart disease affects over 13 million patients, with an earlier onset and faster growth rate in rural areas than urban regions. However, only approximately 40% of acute MI patients receive reperfusion therapy within 12 h of symptom onset, indicating significant treatment delays. Therefore, beyond improving emergency medical systems, developing novel therapeutic strategies that promote myocardial tissue repair and enhance long-term prognosis has become an urgent clinical need [3].

A relatively well-established emergency care system has been developed to address MI, a global health crisis. Reperfusion strategies, exemplified by primary percutaneous coronary intervention (PCI), have become the gold standard of treatment. By rapidly restoring blood flow, these strategies have been continuously improved in application rates under global initiatives [4]. However, clinical practice indicates that successful revascularization is far from the therapeutic endpoint. Current approaches face significant bottlenecks in promoting substantial myocardial regeneration and preventing ventricular remodeling [5,6,7]. While pharmacological therapies (such as antiplatelet agents and statins) can slow disease progression, they cannot regenerate necrotic myocardial cells [8]. Revascularization procedures also encounter numerous limitations, primarily due to risks such as reperfusion injury, restenosis, and late thrombosis. Their fundamental role is to restore “pipeline” patency rather than repair the damaged “engine” (myocardial tissue) [9]. Stem cell therapies were once viewed as promising, but they have failed to meet clinical translation expectations due to low cell survival in the harsh infarct microenvironment, poor homing efficiency, and potential safety risks [10,11]. Therefore, advancing current treatment approaches hinges on developing novel biomaterial platforms that can actively improve the local cardiac microenvironment, provide mechanical support, and efficiently deliver therapeutic agents.

### 1.2. Rationale for SF as a Therapeutic Candidate

To overcome the limitations of current MI treatments, tissue engineering and biomaterials science offer a novel solution. The core challenge lies in developing an ideal biomaterial that highly mimics the natural extracellular matrix of cardiomyocytes, possesses excellent biocompatibility, exhibits precisely controllable mechanical properties and degradation characteristics, and can actively guide tissue toward regeneration rather than fibrosis. Against this backdrop, SF derived from natural silk emerges as a highly promising material for myocardial repair due to its exceptional comprehensive properties [12].

The success of SF stems primarily from its near-perfect physicochemical properties as a biocompatible scaffold, which can be precisely engineered through advanced processing techniques [13]. SF exhibits extremely low immunogenicity, and its degradation products are amino acids metabolized by the body, ensuring high safety. By adjusting the crystallinity of its β-sheet structure, its degradation time can be precisely tuned from weeks to months, ideally matching the dynamic process of myocardial regeneration [2]. Furthermore, SF uniquely combines high strength with flexibility, and its elastic modulus can be tuned to match that of myocardial tissue [14,15]. This enables it to provide critical mechanical support to fragile post-infarction ventricular walls, effectively limiting pathological ventricular dilation while accommodating the heart’s ongoing contractions [16]. SF can also be processed into diverse forms to address varying clinical needs, from in situ hydrogels for minimally invasive injection to precision cardiac patches with biomimetic vascular networks [2,17]. Its versatile preparations demonstrate robust potential for clinical translation.

Recent studies indicate that SF actively guides myocardial repair not only as a physical scaffold but also as a multifunctional platform with anti-inflammatory, antioxidant, and pro-angiogenic activities. Regarding anti-inflammatory effects, SF materials can regulate immune responses [18]. For instance, SF microneedles loaded with specific peptide segments can mitigate excessive macrophage inflammation and promote their transition to a reparative phenotype [1]. Chemically modified SF nanoparticles can even directly reprogram pro-inflammatory macrophages to an anti-inflammatory phenotype [19]. SF and its derivatives also exhibit intrinsic potential to scavenge reactive oxygen species (ROS) [20]. Recent computational simulations have identified novel antioxidant peptides derived from SF, confirming their efficacy in countering oxidative damage associated with cardiovascular diseases [21]. The photo-crosslinked SF hydrogel can significantly promote the migration, proliferation and tube formation of human umbilical vein endothelial cells in vitro, and effectively induce neovascularization in vivo, exhibiting excellent pro-angiogenic capacity [22]. Salt-leached SF scaffolds themselves possess pro-angiogenic activity, capable of inducing angiogenesis and vascular ingrowth; seeding with human adipose-derived stem cells significantly advances the onset of angiogenesis [23]. 

### 1.3. Objectives

This review aims to systematically evaluate recent advances in SF-based repair strategies for MI. It first elucidates SF’s unique physicochemical properties and intrinsic biological activity as an ideal biomaterial platform, focusing on two main application formats: cardiac patches and injectable hydrogels. Subsequently, this review critically analyzes the mechanisms of action and translational potential of these strategies. The core focus lies in examining how SF materials proactively engage with the pathological microenvironment following MI. This includes, but is not limited to, modulating macrophage polarization to reduce inflammation, scavenging ROS to counter oxidative stress, promoting angiogenesis to improve perfusion, and suppressing excessive fibroblast activation to delay fibrosis. Simultaneously, by integrating and comparing existing preclinical data, we objectively evaluate the efficacy and safety of various SF-based therapeutic approaches. We also discuss practical challenges such as standardized production, long-term biocompatibility, integration with host tissues, and large-scale clinical translation.

## 2. Mechanisms of SF in Mitigating MI Pathology

### 2.1. Regulation of Inflammatory Response

Excessive and uncontrolled inflammatory responses following MI represent key pathological mechanisms leading to secondary myocardial injury and adverse ventricular remodeling. SF-based preparations hold significant value, as they can actively intervene and precisely modulate this complex immune process, thereby transforming a destructive inflammatory microenvironment into a regenerative environment conducive to repair [24]. The anti-inflammatory effects of SF are primarily manifested through the effective suppression of core inflammatory signaling pathways. Studies demonstrate that SF-based scaffolds or other drug delivery systems significantly downregulate the expression levels of key pro-inflammatory cytokines, such as tumor necrosis factor-α (TNF-α), interleukin-6 (IL-6), and interleukin-1β (IL-1β), both in the infarct region and the systemic circulation [19]. This inhibitory effect stems not only from SF’s inherent anti-inflammatory properties but also from its role as an excellent carrier. It enables sustained local release of small-molecule anti-inflammatory drugs, specific antibodies, or bioactive peptides, maintaining persistent drug concentrations at the injury site. This precisely blocks the inflammatory cascade, reducing inflammation-induced cardiomyocyte apoptosis and necrosis.

At the cellular level, SF’s regulatory effects are particularly pronounced on immune cells, including early-infiltrating neutrophils and subsequently dominant macrophages. During the acute phase of MI, neutrophils are the first immune cells recruited to the injury site. Their classical N1 phenotype releases large amounts of ROS and proteases, exacerbating tissue damage. SF-based preparations may mitigate this destructive effect by modulating the microenvironment, thereby promoting neutrophil polarization toward the more reparative N2 phenotype [19]. Further research has focused on macrophages. SF can effectively drive macrophages from pro-inflammatory, destructive M1 phenotypes toward anti-inflammatory, repair-promoting M2 phenotypes, depending on its physical topology, degradation products, and loaded bioactive factors [25]. For example, chemically modified SF nanoparticles have been shown to effectively reprogram M1 macrophages into M2 macrophages [19]. Concurrently, SF-based microneedle patch systems guide macrophage functional shifts by delivering immunomodulatory peptides [26]. This regulation of macrophage polarization not only suppresses inflammation at its source but also actively harnesses M2 macrophages to secrete bioactive substances like vascular endothelial growth factor (VEGF), directly initiating tissue repair and angiogenesis programs [2].

### 2.2. Antioxidant Effects

During MI treatment, restoring blood supply to the ischemic area (reperfusion) is essentially a double-edged sword. While salvaging dying myocardial tissue, it also triggers explosive production of ROS, leading to severe oxidative stress. This process is central to exacerbating cellular damage, triggering programmed cell death, and ultimately increasing the infarct size. Notably, SF’s inherent antioxidant bioactivities furnish a crucial pharmacological basis for its therapeutic interventions. The antioxidant potential of SF serves as its primary protective defense. The abundance of functional groups within its molecular backbone, such as hydroxyl groups, enables it to act as an electron donor, directly neutralizing excess ROS like superoxide anions and hydroxyl radicals [27]. This mitigates oxidative damage to myocardial cell lipids, proteins, and genetic material. Cutting-edge bioinformatics analysis combined with in vitro experiments has identified several characteristic peptide segments within SF’s primary structure that exhibit highly efficient radical scavenging capabilities [21]. This discovery not only reveals the molecular basis of SF’s antioxidant activity but also indicates that SF itself can serve as an endogenous antioxidant source. When processed into hydrogels or nanofibers and implanted into MI sites, SF establishes a sustained local antioxidant microenvironment, providing long-term protection against oxidative damage and paving the way for the survival of vulnerable cardiomyocytes.

### 2.3. Anti-Apoptotic Effects

Cardiac cell apoptosis is a key factor in infarct size expansion and ventricular function deterioration, primarily mediated through the mitochondrial pathway. The literature suggested that SF may affect cardiomyocyte apoptosis through multiple pathways. SF-based hydrogel, combined with stromal cell-derived factor-1 alpha and oxygen-releasing microparticles, reduces cardiomyocyte apoptosis post-MI by alleviating hypoxia/inflammation, lowering TUNEL/HIF-1α expression, and enhancing cell survival [16]. In contrast to free glial cell line-derived neurotrophic factor (GDNF) solution, SF-based scaffolds loaded with GDNF exhibited higher anti-apoptotic efficiency, as reflected by increased levels of the Bcl-2 and decreased levels of Bax. This enhancement may be attributed to the synergistic effect between the active factor and the scaffold biomaterial, as well as the sustained and stable release of GDNF at the injured site [28]. SF could alleviate burn-induced tissue injury and apoptosis in a rat model of burn-induced lung injury by increasing the Bcl-2/Bax ratio and inactivating caspase-3 [29]. Flow cytometric analysis using Annexin V and 7-AAD showed that SF hydrolysate suppressed high glucose-induced early apoptosis in RIN-5F cells [30]. SF as a carrier in PDA/CUR@SF microspheres, combined with curcumin and polydopamine, inhibits islet β-cell apoptosis by upregulating Bcl2, downregulating Bax/Caspase3, and relieving oxidative/inflammatory stress [31].

### 2.4. Promotion of Angiogenesis

Following MI, the ischemic zone undergoes myocardial cell necrosis due to interrupted blood flow, accompanied by severe destruction of the vascular network. Restoring blood supply is a critical step in the repair process, and angiogenesis is essential for supplying oxygen and nutrients to surviving myocardium and supporting the viability of newly formed tissue. SF not only serves as a physical scaffold to guide vascular growth but also actively promotes angiogenesis through multiple biological mechanisms [32].

VEGF is a central regulator of angiogenesis. Studies indicate that SF or its degradation products possess the potential to induce endogenous VEGF upregulation. When SF scaffolds are implanted into infarcted myocardium, their excellent biocompatibility mitigates excessive local inflammation, creating a microenvironment conducive to repair. This indirectly promotes VEGF secretion by cardiomyocytes, fibroblasts, and recruited immune cells [33]. More importantly, SF serves as an excellent carrier for controlled and sustained release of pro-angiogenic factors such as VEGF and basic fibroblast growth factor (bFGF) [34]. This continuous local growth factor release effectively and persistently stimulates endothelial cell migration, proliferation, and luminal formation, establishing new capillary networks in both the infarct border zone and core area.

Beyond stimulating in situ angiogenesis, SF also systemically mobilizes and recruits circulating endothelial progenitor cells (EPCs) to the site of cardiac injury. EPCs possess the potential to differentiate into mature endothelial cells and participate in vascular formation [35]. The three-dimensional porous structure of SF preparations provides ideal physical support for EPC adhesion, colonization, and differentiation [36]. Simultaneously, SF loaded with chemokines (e.g., SDF-1α) or its modulated local microenvironment can actively recruit EPCs to home within the infarct area. Upon arrival, EPCs are activated by the combined effects of the SF scaffold and locally released growth factors, exhibiting significantly enhanced proliferation and angiogenic capabilities. This further accelerates the maturation and functionalization of new blood vessels, effectively improving perfusion in the infarct region [37].

### 2.5. Modulation of Cardiac Remodeling

Excessive fibrosis is a primary cause of reduced cardiac compliance and abnormal electrical conduction. Transforming growth factor-β (TGF-β) is a core driver of myocardial fibroblast activation and excessive extracellular matrix deposition, with its downstream Smad signaling pathway serving as a key transmission route [38]. SF intervention effectively modulates this pathway. On one hand, SF’s anti-inflammatory properties reduce TGF-β secretion by early inflammatory cells. On the other hand, SF serves as a delivery platform for inhibitors (e.g., small-molecule drugs, specific miRNAs, or neutralizing antibodies) that directly target and block the TGF-β/Smad signaling pathway [39]. This inhibits the transformation of fibroblasts into hyperactive myofibroblasts, reducing excessive collagen fiber synthesis and abnormal accumulation. Consequently, scar stiffness is mitigated and ventricular dilation is delayed.

SF exerts both direct and indirect effects in maintaining and improving overall cardiac pump function. Indirect effects stem from its promotion of angiogenesis and inhibition of fibrosis, creating a microenvironment more conducive to surviving myocardium and preserving normal ventricular geometry. Direct effects manifest as SF-based preparations (e.g., conductive patches) providing mechanical support to infarct regions, preventing wall thinning and aneurysm formation, reducing wall stress, and thereby improving global cardiac mechanics. When incorporated into cardiac film or used to encapsulate cardiomyocytes, SF enhances cell adhesion, alignment, and intercellular junctions, and has been demonstrated to significantly improve cardiomyocyte contractility [40]. Furthermore, by delivering protective substances (e.g., anti-apoptotic factors) or serving as a stem cell carrier, SF directly promotes contractility recovery by protecting compromised cardiomyocytes and replenishing functional cells [41].

**Figure 1:**
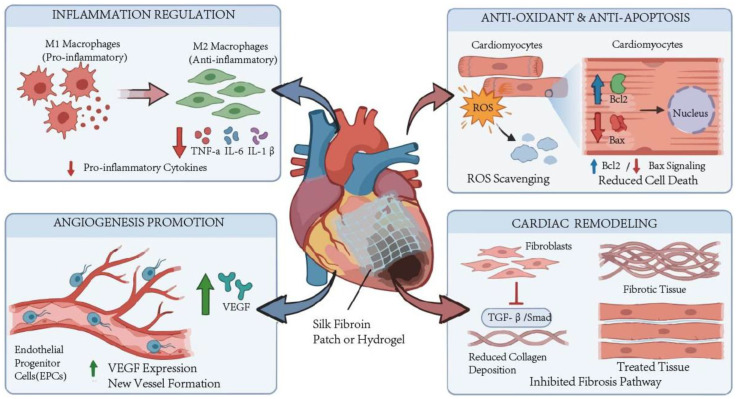
Mechanisms of SF in mitigating MI pathology. (SF-based preparations improve MI via multiple mechanisms, including anti-inflammation, antioxidation, anti-apoptosis, angiogenesis promotion, and cardiac remodeling modulation. ↓ indicates downregulated, and ↑ indicates upregulated).

In summary, SF preparations can improve the state of MI through multiple mechanisms, mainly including regulation of inflammatory response, antioxidant effects, anti-apoptotic effects, promotion of angiogenesis, and modulation of cardiac remodeling. The summary of these mechanisms is shown in Figure 1.

## 3. Applications of SF in MI Therapy

A large number of cardiomyocytes in the MI area die, leading to issues such as ventricular wall thinning and adverse remodeling. Local microenvironmental disturbances can severely impede myocardial repair processes and affect cardiac function recovery. Single therapeutic approaches often fail to precisely target the infarcted region and cannot sustain treatment effects over the long term, making it difficult to achieve ideal myocardial repair outcomes. Biomaterials, as critical carriers for delivering therapeutic components and modulating local microenvironments, play a pivotal role in MI treatment. SF, with its excellent biocompatibility and degradability, has emerged as an ideal choice for constructing myocardial repair materials. Consequently, efforts have been dedicated to combining SF with various functional components to develop preparations tailored for MI treatment. Currently, SF-based myocardial repair preparations have been widely developed and extensively studied, including cardiac patches and injectable hydrogels. These SF-based preparations have demonstrated significant efficacy in inhibiting ventricular adverse remodeling and combating local inflammation. Additionally, they have been proven to promote cardiomyocyte survival and vascular regeneration, effectively accelerating myocardial repair processes and improving cardiac function.

### 3.1. SF-Based Cardiac Patches

SF-based patches have established a diversified application system with their precisely controllable mechanical properties, excellent biocompatibility, and flexible structural modification potential. These patches are designed to address the four core requirements of MI repair, including mechanical support, cell adhesion, active regeneration, and electrical signal conduction. These patches integrate the biocompatibility of natural biomaterials with targeted functional modifications through differentiated design, providing multidimensional and personalized solutions for MI treatment. The following section systematically elucidates their application mechanisms and research progress based on material functional characteristics, highlighting their core value in myocardial repair through the latest literature findings.

#### 3.1.1. SF Composite Patch Applied as a Passive Mechanical Support

SF composite patches are core passive mechanical support materials for cardiac repair after MI. Their core advantages stem from the excellent biocompatibility, controllable degradability, and mechanical toughness of SF itself. Through self-structural regulation and multi-component composite modification, they achieve precise matching with the mechanical environment of the damaged myocardium, thereby effectively inhibiting adverse ventricular remodeling, resisting cyclic stress on the ventricular wall, preventing wall thinning and ventricular dilation, creating a stable microenvironment for myocardial regeneration, and thus reducing the risk of cardiac function deterioration [42,43].

The unique secondary structure of SF endows it with a regulable mechanical basis, among which the content of β-sheets is a key variable determining the stiffness of the material. Studies by Lo et al. have confirmed that the β-sheet content of SF can be controlled between 20% and 44% through methods such as salting out and ethanol treatment, thereby constructing SF/poly-ε-caprolactone (PCL) patches with gradient stiffness [44]. This regulatory strategy enables the SP20/30 patch with low β-sheet content to simulate the flexible environment of healthy myocardium (approximately 10–20 kPa), while the SP44 patch with high β-sheet content has the ability to resist high stress (above 200 kPa) in the infarcted area. Studies on layered scaffolds by Mehrotra et al. further verified this point. Young’s modulus of ethanol-induced non-silkworm SF scaffolds can reach 93.1 ± 6.8 kPa, and they can withstand the dynamic cyclic strain caused by heartbeats, demonstrating the potential of SF in maintaining ventricular mechanical stability [45].

**Figure 2:**
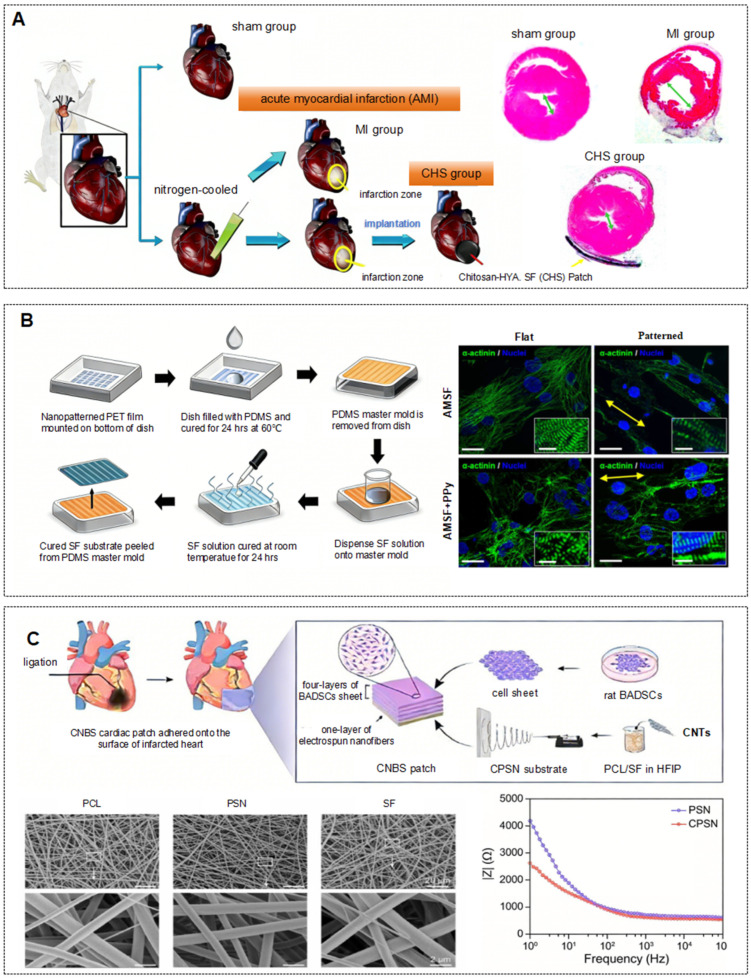
Representative SF-based cardiac patches for MI repair. (**A**) Chitosan-hyaluronic acid/SF composite patch inhibits chamber dilation in rat MI models, increasing the thickness of their walls (Green arrows indicates the inner diameters of the left ventricular.) [48]. (**B**) Capillary force lithography creates anisotropic nanotopography on SF substrates, yielding SF-PPy composite patches that stably enhance cardiomyocyte adhesion and alignment (Yellow arrows indicate the direction of the nanopattern. Scale bars: 25 m; inset scale bars: 10 m.) [49]. (**C**) Cardiac patches composed of brown adipose-derived stem cell sheets and electrospun polycaprolactone/SF nanofibers incorporated with conductive carbon nanotubes enhance cardiac repair in rat MI models [50].

On this basis, the composite modification strategy further expands the mechanical boundary of SF. Bhaarathy et al. successfully reduced the elastic modulus to 7.0 ± 0.9 MPa by combining SF with poly(l-lactic acid)-co-poly (ε-caprolactone) (PLACL) and aloe vera (AV). This flexible characteristic, combined with a high porosity of 94%, effectively balanced the conflict between mechanical support and nutrient permeability [46]. By introducing the superparamagnetic iron oxide nanoparticles SPION-casein core–shell structure, Nazari et al. achieved a maximum tensile strength of 38.4 MPa for SF nanofibers, significantly enhancing the material’s resistance to ventricular wall tension [47]. As shown in Figure 2A, the chitosan-hyaluronic acid/SF composite patch developed by Chi et al. achieves stable support through multi-component synergy while reducing mechanical mismatch at the interface [48].

This precise mechanical adaptation ultimately translated into significant anti-remodeling effects in vivo. Experimental data from Chi et al. demonstrated that after 8 weeks of implantation of the composite patch, the left ventricular diameter in MI rats decreased from 5.92 mm to 4.27 mm, with effective restoration of ventricular wall thickness, confirming its direct role in inhibiting chamber dilation [48]. Meanwhile, the study by Bhaarathy et al. demonstrated that flexible PLACL/SF/AV patches avoided the cell growth inhibition caused by the excessive rigidity of pure polymer scaffolds, achieving a balance between rigid support and soft repair [46].

In summary, the mechanical support function of SF follows a complete logic from microstructural regulation to macroscopic composite reinforcement, ultimately achieving in vivo anti-remodeling. The core lies in achieving precise matching between material properties and the heterogeneous mechanical environment of the post-MI myocardium through diverse technical approaches, which is the key factor enabling SF materials to effectively support cardiac repair.

#### 3.1.2. Biofunctionalized SF Patches with Improved Tissue-Adhesive Property

The core role of SF in enhancing myocardial cell adhesion by biofunctionalized agents stems from the synergistic effects of its natural structural properties and functional modifications. By providing a fundamental adhesion platform, introducing specific binding sites, optimizing the physical microenvironment, and activating intracellular signaling pathways, SF multidimensionally strengthens the interaction between myocardial cells and the scaffold, thereby laying a critical foundation for cell colonization and tissue repair following MI. The natural structure and biocompatibility of SF serve as the fundamental basis for myocardial cell adhesion. SF’s amino acid composition is rich in residues like tyrosine and alanine, which can modulate surface hydrophilicity by regulating secondary structures (e.g., β-sheet content), providing a gentle physical adhesion interface for myocardial cells [51].

Functional modification introduces specific ligands, and the target adhesion mediated by receptor–ligand interaction is the core mechanism for enhancing adhesion efficiency. SF itself lacks specific cell-binding sites and requires chemical modification or complex modification to introduce targeting molecules, forming specific binding with myocardial cell surface receptors. Gotoh et al. covalently conjugated GlcNAc to SF to prepare SF-GlcNAc conjugates, whose surface-exposed β-GlcNAc residues can specifically recognize desmin on myocardial cell surfaces. The binding affinity between these conjugates and desmin was significantly higher than that of unmodified SF. This receptor–ligand interaction enhances the adhesion stability of human cardiomyocytes [52]. Liu et al. modified SF with extracellular matrix (ECM) derived from cardiac fibroblasts. The natural ligands in ECM, such as collagen types I, III, IV, fibronectin, and laminin, can bind to integrins on the surfaces of cardiomyocytes and stem cells, mediating specific adhesion. This resulted in a 1.5-fold increase in the adhesion number of brown adipose stem cells (BASCs) compared with the pure SF group, with a significant expansion of the adhesion area [53].

The optimization of the physical microenvironment enhances the stability of myocardial cell attachment through mechanical anchoring effects. SF can simulate the physical microenvironment of the natural ECM by modulating topological structures, carrier morphology, and other physical properties, thereby increasing the contact area between cells and the formulation and strengthening mechanical anchoring. As shown in Figure 2B, Tsui et al. employed capillary force lithography to construct nanoscale ridges and grooves on the surface of SF in SF-PPy composite patches, mimicking the fiber arrangement of the natural myocardial ECM. This guided the directional attachment of human pluripotent stem cell-derived cardiomyocytes, increased the contact area between cells and the carrier, and resulted in the directional alignment of cardiomyocytes, significantly improving attachment stability [49]. The SF methacryloyl microneedles loaded with Tc peptide drugs (SilMA MNs) prepared by Wang et al. exhibit both excellent toughness and tissue adhesion. The array structure of the microneedles provides multiple anchoring sites for cells, reducing detachment. The microneedles can stably remain on the myocardial tissue surface for over 24 h, offering a physical guarantee for sustained attachment of cardiomyocytes [1].

In summary, the natural biocompatibility of SF provides an initial attachment foundation, functional modifications introduce specific ligands to mediate targeted binding, and physical microenvironment optimization enhances mechanical anchoring. These mechanisms work synergistically, enabling SF-based agents to effectively overcome the limitation of pure SF lacking specific binding sites, significantly improving the efficiency and stability of myocardial cell attachment, thereby creating conditions for subsequent cell proliferation, differentiation, and tissue repair. In the future, by precisely regulating the modification methods, physical structure, and signaling pathway targeting of SF, its efficacy in enhancing myocardial cell attachment can be further optimized, promoting clinical translation.

#### 3.1.3. Cell-Laden SF Patches for Myocardial Tissue Regeneration

Stem cell therapy offers promising potential for active regeneration in MI repair. However, traditional direct stem cell injection faces core challenges such as low cell survival rates, poor colonization efficiency, and rapid clearance by the circulatory system, which severely limit therapeutic efficacy. The core value of SF-loaded cell patches lies in leveraging SF’s superior biocompatibility and structural plasticity. Through stem cell-mediated active regeneration and paracrine signaling, these patches alleviate functional cell deficits post-MI, modulate the local microenvironment, and achieve a critical upgrade from structural support to active repair.

The main active regeneration mechanism is that SF constructs an optimal microenvironment for cells, guiding stem cells to differentiate directionally into cardiomyocyte-like cells. Cetin et al. seeded human adipose-derived mesenchymal stem cells (hAD-MSCs) into a 4% concentration 3D SF scaffold (porosity approximately 40%). The porous structure of SF provides sufficient colonization space, while the mechanical properties of β-sheet structures match the contractile requirements of myocardium, supporting sustained cell proliferation. In addition, the SF scaffold induced high expression of myocardial markers such as α-actin and troponin I [54]. As shown in Figure 2C, Wei et al. constructed carbon nanotubes (CNTs) containing electrospun polycaprolactone/silk fibroin nanofibers (CPSN) loaded with brown adipose-derived stem cells (BADSCs). The biocompatibility of SF ensured cell survival, while the conductivity conferred by CNTs synergized with the structural support of SF to promote cell orientation and differentiation. The BADSCs successfully expressed myocardial-specific markers such as cTnT and MEF2C [50]. After inoculation with bone marrow mesenchymal stem cells (BMSCs), Chi et al. demonstrated that the synergistic effect of SF and hyaluronic acid enhanced bioactivity, promoting the differentiation of BMSCs into cardiomyocyte-like cells that express troponin T, with further stabilization of differentiation achieved through interaction with the SF matrix [55].

The paracrine signaling mechanism leverages the structural and modification advantages of SF to enhance cell retention, enabling loaded cells to continuously secrete bioactive factors for microenvironment regulation. Chen et al. employed SF/chitosan modified nanofiber patches loaded with adipose tissue-derived mesenchymal stem cells (AD-MSCs). The combined modification of SF and chitosan significantly improved cell adhesion and substantially increased cell retention. The secreted factors downregulated fibrosis markers such as TGF-β1 and P-smad3, resulting in a nearly twofold increase in left ventricular ejection fraction compared with the MI group [56]. In Chi’s study, BMSCs secreted VEGF, bFGF, and hepatocyte growth factor (HGF) on SF/hyaluronic acid patches, leading to a more than 6-fold increase in vascular density in the infarcted area compared with the MI group [55]. Du et al. transplanted c-kit^+^ bone marrow cells into SF/poly(L-lactic acid-co-ε-caprolactone) scaffolds. The addition of SF improved the hydrophilicity of the carrier (contact angle decreased from 108.8° to 86.9°), reduced the release of creatine kinase MB (CK-MB), and decreased the infarct area by more than 30% [57]. In Wei’s CPSN-BADSCs sheets, SF ensures cell survival and functional performance. Through paracrine regulation, the cells induce macrophage polarization toward the M2 phenotype (with elevated CD163 expression) and secrete anti-inflammatory factors such as IL-10, significantly reducing myocardial apoptosis [50].

As an important carrier, SF exhibits structural plasticity and multifunctional modification capabilities that precisely meet cellular requirements. 3D SF scaffolds provide ample implantation space; SF/chitosan nanofibers enhance cell adhesion through composite modification; SF/hyaluronic acid composite system improves bioactivity; and blends of SF with synthetic polymers optimize mechanical properties and hydrophilicity. Cellular activity endows the patch with active repair capacity, and the synergistic interaction between the two forms a “regenerative cell replenishment and paracrine microenvironment modulation” repair loop, effectively overcoming the limitations of traditional stem cell therapies.

#### 3.1.4. Electroconductive SF Patches

Necrosis of myocardial cells in the infarcted area after MI leads to disruption of electrical signal conduction and disordered intercellular electrical coupling, which is a key factor in the deterioration of cardiac function. The core value of SF conductive patches lies in their ability to leverage the excellent biocompatibility and structural plasticity of SF as a foundation. By integrating conductive agents such as reduced graphene oxide (rGO), CNTs, polypyrrole (PPy), and polyaniline (PANi), these patches are endowed with myocardial-adapted conductive properties. This not only enhances electrical signal conduction within the infarcted region but also modulates the myocardial microenvironment, thereby achieving synergistic therapeutic effects in electrical restoration, structural remodeling, and cardiac functional recovery.

The composite of SF with conductive agents enhances patch conductivity, bridging the electrical signal conduction gap in the infarcted region, while SF itself provides critical support for the uniform dispersion of conductive components and cell colonization. Liang et al. fabricated encapsulated nanofibers by co-spinning PPy and SF. When the SF concentration was 7% and the PPy:SF ratio was 30:70, the patch conductivity reached 0.52 mS/cm, precisely matching the electrical properties of native myocardium. Neonatal rat cardiomyocytes (NRCMs) exhibited anisotropic alignment on the surface, with significantly improved calcium transient synchronicity, demonstrating the facilitation of synchronous electrical signal transmission by conductive properties [58]. Tsui et al. first acid-modified SF (AMSF) to introduce sulfonic acid groups, then recombined it with PPy to achieve a patch conductance of approximately 1 S/cm. The expression levels of gap junction protein Cx43 and sodium channel gene SCN5A increased by 2-fold and 1.8-fold, respectively, compared to the pure SF group. The dual effects of enhanced sodium channel function and upregulated gap junction protein expression accelerated intercellular electrical signal transmission [49]. Tufan et al. fabricated a porous scaffold by combining carbon nanofibers (CNF) with SF, achieving a conductivity of 0.021 S/cm that matches myocardial tissue conductivity. This scaffold not only supports the differentiation of induced pluripotent stem cells (iPSCs) into cardiomyocytes (upregulation of TNNT2 and NKX2.5 genes) but also harvests myocardial motion energy via triboelectric nanogenerator effects, thereby providing additional assurance for electrical signal conduction [59].

Conductive SF patches can also optimize the microenvironment of the infarcted area, removing physical and biochemical barriers to electrical signal conduction. The rGO/SF nanofiber patch developed by Feng et al. not only enhances conductivity through rGO but also downregulates the Yap/Taz-TGFβ1/Smads signaling pathway, reducing myocardial fibrosis (the collagen volume fraction was decreased by 23.6% compared to the MI group) and simultaneously decreasing myocardial stiffness (Young’s modulus of the long axis decreased from 18.61 kPa to 7.63 kPa), thereby establishing a favorable physical foundation for electrical signal conduction [39]. The COL-SF/PANi conductive patch developed by Leite et al. not only confers electrical activity but also modulates antioxidant markers (e.g., elevated CAT activity), thereby reducing oxidative stress interference with electrical signal conduction. This enhances the electrical coordination of myocardial cells in the infarcted area, resulting in a significant improvement in left ventricular ejection fraction (LVEF) compared to the MI control group [60]. The CPSN-BADSCs layer in Wei’s system can also modulate macrophage polarization to M2-type through paracrine regulation, thereby reducing inflammatory responses and indirectly improving the electrical conduction microenvironment, resulting in more stable electrical signal transmission [50].

In summary, SF-based patches address the core requirements of structural stability, cell adhesion, active regeneration, and electrical signal integration in MI repair through four design approaches: passive mechanical support, biofunctional modification, cell loading, and conductive modification. This establishes a multidimensional and synergistic therapeutic system. All patch types have demonstrated significant efficacy through in vitro and in vivo experiments, providing diverse options for precision treatment of MI. Leveraging the superior biocompatibility and scalable production advantages of SF materials, these patches exhibit broad prospects in clinical translation, offering novel therapeutic hope for MI patients.

### 3.2. Injectable SF Hydrogels

Ischemia and hypoxia-induced myocardial cell necrosis, ventricular remodeling, and inflammatory microenvironment imbalance are key factors leading to cardiac dysfunction post-MI. Hydrogels, with their three-dimensional network structure, biocompatibility, and functional tailorability, have emerged as important formulations for myocardial repair. Injectable SF hydrogels are delivered minimally invasively to myocardial injury sites, forming a three-dimensional porous network structure that effectively fills infarcted cavities and provides mechanical support. Their high hydrophilicity and microporous structure facilitate cell retention, nutrient diffusion, and metabolic waste clearance, while simultaneously mimicking the natural ECM microenvironment to promote the recruitment and directional differentiation of endogenous precursor cells.

#### 3.2.1. In Situ Gelatin Hydrogels to Provide Mechanical Bulking

The adverse remodeling caused by left ventricular wall thinning and increased wall stress following MI is a key factor in the deterioration of cardiac function. SF in situ gelatin hydrogel not only provides mechanical filling to reduce wall stress but also guides tissue regeneration through controlled degradation. Hu et al. demonstrated that the SF hydrogel not only offers mechanical support but also induces the transformation of quiescent ventricular cardiomyocytes into pacemaker cells (HCN4 positive cells accounted for 18.5%), improving electrical signal conduction and indirectly reducing the risk of electrical remodeling caused by mechanical abnormalities, thereby forming dual mechanical–electrophysiological protection [61]. The Kambe team designed two types of SF hydrogels: unmodified (SF) and peptide-modified (SF + Pep). Both exhibited consistent stiffness but different degradation rates. The SF group formed dense, randomly arranged collagen fibers, which were more resistant to ventricular pressure compared to the sparse, oriented fibers in the SF + Pep group, thereby further consolidating the mechanical support effect [62].

#### 3.2.2. Hydrogels as Cell Carriers

**Figure 3:**
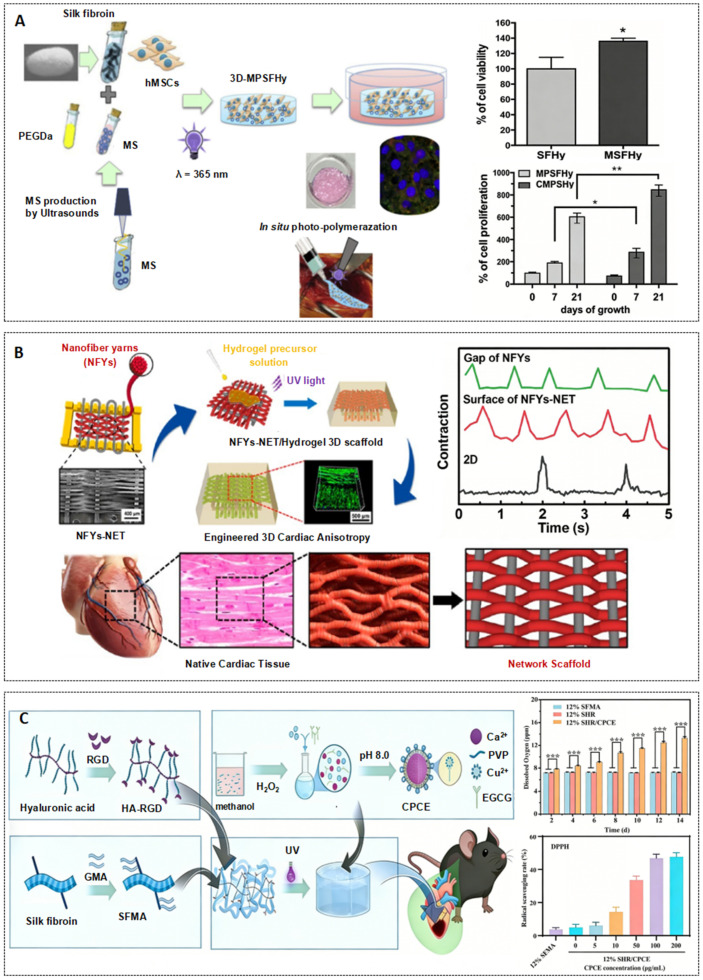
Representative injectable SF Hydrogels for MI repair. (**A**) Injectable silk fibroin hydrogels functionalized with albumin microspheres enhance cardiac mesenchymal stem cells functions in 3D cell culture (* *p* < 0.05, ** *p* < 0.01) [63]. (**B**) A 3D hybrid scaffold composed of polycaprolactone, SF, and CNTs, featuring an aligned conductive nanofiber yarn network within a hydrogel shell, for engineering 3D cardiac anisotropy in cardiac tissue engineering [65]. (**C**) A hydrogel composed of SF methacryloyl, hyaluronic acid-modified RGD peptide, calcium peroxide, and copper-epigallocatechin gallate promotes myocardial repair, reduces fibrosis, enhances angiogenesis, and improves cardiac function (*** *p* < 0.001) [66].

Post-MI cell therapy faces core challenges such as cell loss, low survival rate, and suboptimal functional performance. As cell carriers, hydrogels leverage their advantages of in situ delivery, microenvironmental mimicry, and functional adaptation to directly deliver therapeutic cells to the injury site. This provides support for cell survival, proliferation, and functional differentiation, making them a key strategy for myocardial regeneration therapy. As shown in Figure 3A, Ciocci et al. constructed an SF/polyethylene-glycol-diacrylated composite hydrogel, which exhibited increased porosity after embedding albumin microspheres, resulting in a 36.2% improvement in the viability of loaded cardiac mesenchymal stem cells. Further incorporation of CS led to a 746.3% increase in cell proliferation rate within 21 days, accompanied by high expression of myocardial markers such as α-actin and connexin 43 [63]. Sogol Motallebi Tala Tapeh et al. designed thermosensitive SF/hyaluronic acid/Aloe vera composite hydrogels with an elastic modulus between 68.1 and 74.63 kPa, which matched that of myocardial tissue. The Pgel/AV/SF group exhibited high expression of early myocardial genes such as GATA4 and NKX2.5, while the Pgel/HA/SF group showed upregulation of mature markers, including GJA1 and TNNI3 [64]. Vettori et al. incorporated 1% (*w*/*v*) SF into alginate/gelatin hydrogels. Following 3D bioprinting, the elasticity was enhanced, the survival rate of the encapsulated cardiac spheroids remained stable, the fractional shortening was increased by 41%, and the contractile function was improved [15].

The key to matching the mechanics and structure of hydrogels lies in guiding the maturation of cell functions through dynamic mechanical regulation and directional structural design. Stoppel et al. developed SF/cardiac tissue-derived ECM composite hydrogels, which exhibited time-dependent stiffening, regulated the expression of integrins and the formation of focal adhesions in cardiac fibroblasts, and promoted endogenous cell infiltration and endothelial cell growth after 4 weeks of in vivo transplantation [67]. As shown in Figure 3B, Wu et al. constructed SF/PCL/CNT nanofiber yarn-GelMA hydrogel scaffolds. The yarn network guides the oriented arrangement of cardiomyocytes, increases Cx43 expression, and improves the synchrony of calcium transients. After being combined with endothelial cells, a vascularized myocardial structure formed, and the contraction synchrony was enhanced [65].

These studies confirm that the core advantages of hydrogels as cell carriers are concentrated in three aspects. First, composite modification optimizes the microenvironment for cell adhesion, improving cell survival and differentiation rates. Second, mechanical and structural adaptation guides the maturation of cell functions. Third, targeted delivery and multi-cell synergy enable minimally invasive and precise delivery, as well as vascularized integration. The biocompatibility and structural plasticity of SF ensure the safety and functional adaptability of the carrier, and composite modification and structural design further enhance cell delivery efficiency and therapeutic effects.

#### 3.2.3. Hydrogels for Sustained Drug/Growth Factor Release

Microenvironmental disorders after MI, including hypoxia, oxidative stress, inflammatory imbalance, and insufficient angiogenesis, are the main causes of cardiac function deterioration. SF-based hydrogels loaded with bioactive components can, via their sustained-release properties, precisely regulate the microenvironment of the infarcted area from multiple dimensions, construct a favorable niche for myocardial repair, and thus serve as a key strategy to improve prognosis.

Hydrogels address the key issues of hypoxia and oxidative stress through the synergy of oxygen supply and antioxidant properties. As shown in Figure 3C, Song et al. developed a hydrogel composed of SF methacryloyl, hyaluronic acid-modified RGD peptide (HA-RGD), calcium peroxide (CaO_2_), and copper-epigallocatechin gallate (Cu-EGCG). The hydrolysis of CaO_2_ continuously supplies oxygen to alleviate ischemia, while Cu-EGCG efficiently scavenges ROS, significantly improving the oxidative stress microenvironment. Its three-dimensional network, in synergy with HA-RGD, enhances cell adhesion, laying a cellular foundation for myocardial repair [66]. Although other studies have not yet achieved direct synergistic design of oxygen supply and antioxidant effects, Feng et al.’s SF microspheres/alginate hydrogel can indirectly alleviate hypoxia-induced oxidative damage through the sustained release of IGF-1 [68]. Hua et al.’s SF/hydroxypropyl cellulose hydrogel leverages the superior biocompatibility of SF to mitigate secondary oxidative stress-induced cellular damage [12], which indirectly validates the fundamental supportive role of SF-based hydrogels in oxidative stress regulation.

Hydrogels alleviate inflammatory imbalance and inhibit fibrosis by continuously releasing anti-inflammatory components. Hua et al.’s SF/hydroxypropyl cellulose hydrogel loaded with folic acid-modified extracellular vesicles (FA-EVs) releases bioactive components in a sustained manner. After 28 days of in vivo injection, the expression of transforming growth factor β1 (TGF-β1) was significantly downregulated, the myocardial fibrosis area decreased from 32.99% to 5.49%, and the levels of inflammatory factors were simultaneously decreased [12]. Ni et al. loaded FA-modified exosomes (FA-EXOs) into SF/alginate hydrogels. After sustained release, the expression of TGF-β1 was inhibited, the left ventricular collagen area decreased from 39.35% to 4.04%, and pathological remodeling was effectively blocked [17].

Hydrogels reconstruct the blood supply microenvironment in ischemic areas by continuously releasing pro-angiogenic components. Feng et al.’s SF microspheres/alginate hydrogel loaded with insulin-like growth factor 1 (IGF-1) achieved sustained release for 28 days, promoted the proliferation of H9c2 cells in vitro, upregulated the expression of vascular-related genes in the infarcted area in vivo, reduced infarct size, and significantly improved LVEF [68]. In Song’s hydrogel, Cu^2+^ was slowly released, which in synergy with EGCG promoted the formation of HUVEC tubular structures. The density of CD31-positive blood vessels in vivo was significantly increased, improving nutrient delivery to ischemic areas [66].

Injectable SF hydrogels, with their excellent biocompatibility and functional plasticity, synergistically address issues such as post-MI ventricular remodeling, low cell survival rate, and microenvironmental imbalance through three major strategies: in situ gelation for mechanical support, cell carrier delivery, and sustained release of bioactive substances, thereby providing an efficient platform for myocardial repair. Their minimally invasive delivery property meets clinical requirements. Upon component and structure optimization, they can achieve the synergistic effects of mechanical support, cell regulation, and microenvironmental modulation, significantly improving cardiac function after MI. Existing studies have fully verified their application potential. In the future, by integrating intelligent response mechanisms and optimizing translational processes, injectable SF hydrogels are expected to advance from basic research to clinical application, providing a new strategy for the treatment of MI.

## 4. Current Challenges and Future Perspectives

Despite demonstrating excellent biocompatibility, processability, and potential in diverse MI repair applications, the translation of SF from experimental research to clinical practice remains constrained by a series of fundamental material limitations and translational challenges. These drawbacks also highlight key directions for future research.

A major bottleneck arises during the early stages of SF material preparation. The molecular composition and structural characteristics of SF are susceptible to variations in cultivation environments, climatic conditions, and processing batches [69]. Batch-to-batch variations in natural silk result in inconsistent molecular weight distributions and β-sheet ratios, ultimately compromising the material’s mechanical properties and reproducibility [70,71]. Therefore, establishing standardized raw material grading, processing protocols, and structural characterization systems, combined with quality assurance through molecular fingerprinting or high-throughput characterization techniques, is fundamental to producing reliable clinical-grade SF [72]. From the preparation perspective, meaningful standardization requires transitioning from reliance on natural silk alone to a scientifically controlled preparation system, involving strict regulation of raw material selection, process parameters, and structural characterization [73]. Furthermore, modern machine learning holds significant potential for quality control. By enabling high-throughput quantitative assessment of key parameters such as molecular weight distribution, β-sheet ratio, and purity, it promises to substantially reduce batch-to-batch variability and enhance preparation consistency [74].

Processing and manufacturing stages also present challenges. Conventional dissolution and degumming methods often employ alkaline or enzymatic solutions, leading to degradation of fibrin peptide chains and reduced molecular weight, which compromises mechanical properties and cell-material interactions [14]. Developing mild, non-denaturing extraction techniques, such as enzyme-selective, ionic liquid, or green solvent systems, can remove potential immunogens while preserving molecular integrity. Lactic acid/choline chloride deep eutectic solvents demonstrate superior biocompatibility compared with conventional lithium bromide systems [75,76], and calcium chloride–ethanol–water systems rapidly dissolve high-molecular-weight SF under mild conditions while preserving an extended random coil conformation and minimizing protein damage [77]. Scaling from laboratory to industrial production remains problematic. Electrospinning suffers from limited throughput and structural precision. Melt electrowriting enables precise structures, but SF’s lack of thermoplasticity requires lattice bonding with PCL or similar materials. Solution electrowriting preserves protein structures at room temperature, yet stacking accuracy is constrained by water evaporation. Micro-stereolithography achieves high spatial precision but requires throughput optimization and regulatory compliance. Maintaining fine structures while ensuring manufacturing consistency is critical for the clinical translation of SF [78,79].

Insufficient understanding of long-term in vivo behavior also represents a translational bottleneck. Most studies focus on short-term responses in small animals, resulting in inadequate characterization of degradation kinetics, metabolites, and systemic immune effects [80]. Although SF is generally considered biocompatible, its degradation products (peptides and amino acids) may exert differential effects across different organs or pathological states. Large-animal and chronic animal studies, combined with imaging and metabolomics tracking technologies, are crucial for defining safe degradation windows and guiding scaffold design. Furthermore, the immune response to SF is highly dependent on the local microenvironment. Factors such as stiffness, porosity, and surface chemistry can influence macrophage polarization and foreign body reactions [81]. SF organoid platforms can reproduce the physiological, structural, and functional characteristics of native tissues, enabling controlled long-term studies of degradation, immune, and cellular responses, thereby overcoming limitations of traditional animal models [82,83]. Integrated systemic immune analysis that incorporates both local and systemic responses should become part of the preclinical testing standard.

Translational application remains constrained by material stability and production realities. SF solution stability, storage conditions (currently up to 6 months), and transportation requirements are extremely demanding. Combined with the high cost of purification and processing technologies, these factors severely limit large-scale commercial application [84,85]. Future research should explore formulation engineering, such as developing more stable solid or pre-formulated material formats to enhance storage stability and usability. Simultaneously, issues like complex purification and processing procedures, high costs, and inconsistent quality control standards indicate that subsequent development must concurrently advance production process standardization, establish quality evaluation systems, and implement cost control strategies to improve industrial feasibility. Finally, most existing studies rely on in vitro experiments or small animal models, and discrepancies between in vitro and in vivo degradation behaviors may affect therapeutic efficacy assessments [86]. Future research must therefore systematically validate the safety, efficacy, and functional stability of SF materials using large animal models that more closely mimic clinical pathological conditions, supplemented by long-term follow-up data, to provide reliable evidence for clinical translation [87].

Overall, the future development of SF-based preparations will no longer be confined to enhancing individual properties. Instead, it will involve coordinated advancement across multiple fronts, such as standardizing material sources, achieving controllable degradation, systematically validating biological safety, optimizing functional biomimicry, and refining the translational system. This multifaceted approach will facilitate a smooth transition from experimental exploration to being clinically implementable.

## 5. Conclusions

Developing SF-based preparations with excellent biocompatibility, degradability, and stable tissue integration has become a key research strategy in MI repair, aimed at addressing the complex and dynamically changing pathological microenvironment following MI. Pathological alterations in local myocardial tissue after MI, including persistently activated inflammatory responses, elevated oxidative stress, impaired vascular structures, and weakened myocardial electrical signal conduction, provide a clear biological basis for constructing engineered SF preparations with tunable structural properties and versatile functionalization. SF-based cardiac patches and injectable hydrogels can directly target the infarcted microenvironment through localized delivery or tissue coverage. These preparations provide mechanical support to the injured myocardium while modulating inflammation, alleviating oxidative stress, and promoting angiogenesis, thus exerting beneficial effects on attenuating ventricular remodeling and restoring cardiac function. In recent years, with the continuous advancement of material functionalization strategies, SF-based preparations loaded with cells, bioactive molecules, or conductive components have exhibited further multifaceted regulatory functions, including supporting cardiomyocyte survival, enhancing electrical signal conduction, and promoting tissue integration, highlighting their potential for comprehensive intervention in MI. Although these studies have achieved promising outcomes in various preclinical models, challenges still exist in further optimizing SF material properties, evaluating long-term biocompatibility and degradation behavior, and achieving precise control over therapeutic factor delivery. Continued multidisciplinary research is essential to systematically advance SF-based myocardial repair strategies from experimental studies towards clinical translation. Looking ahead, the integration of advanced biomanufacturing techniques, smart responsive designs, and combined therapeutic strategies holds promise for expanding the application scope of SF-based preparations in MI repair.

## Data Availability

No new data were created or analyzed in this study. Data sharing is not applicable to this article.

## References

[1] Wang X., Zhang J., Hui J. (2025). Peptide-Laden Silk Fibroin Microneedles Modulate Inflammation and Promote Myocardial Repair In Vitro. Macromol. Biosci..

[2] Moradi A., Nouri S., Ulag S., Gunduz O. (2024). On-demand release of fucoidan from 3D-printed cardiac scaffolds based on chitosan/silk fibroin/polyaniline. Mater. Lett..

[3] Cheng N., Luo Q., Yang Y., Shao N., Nie T., Deng X., Chen J., Zhang S., Huang Y., Hu K. (2025). Injectable pH Responsive Conductive Hydrogel for Intelligent Delivery of Metformin and Exosomes to Enhance Cardiac Repair after Myocardial Ischemia-Reperfusion Injury. Adv. Sci..

[4] Herrera C.J., Levenson B.J., Natcheva A., Lucca A.C., Olsson K., Miki K., Fong A., Jollis J.G., McCormick A., Wilson B.H. (2025). Improving STEMI Management Internationally: Initial Report of the American College of Cardiology-Global Heart Attack Treatment Initiative. JACC Adv..

[5] Xu S., Zhang R., Jin S., Luo H., Hou Y., He S., Shi Z., Zhao R., Chen Z., Wang B. (2025). The activation of catecholamine neurons in the rostral ventrolateral medulla drives ventricular remodeling after myocardial ischemia/reperfusion injury. Basic Res. Cardiol..

[6] Bois A., Grandela C., Gallant J., Mummery C., Menasché P. (2025). Revitalizing the heart: Strategies and tools for cardiomyocyte regeneration post-myocardial infarction. npj Regen. Med..

[7] Yao L., An H., Fan C., Lan Q., Zhong H., Zhang Y., Zhou L., Hao P. (2026). Injectable BMSC-Based Extracellular Matrix-Mimicking Microtissue for Myocardial Infarction Repair. Adv. Sci..

[8] Gao S., Li D., Wang B., Zhang H., Chen L. (2025). Two promising approaches in the treatment of myocardial infarction: Stem cells and gene therapy. Front. Cardiovasc. Med..

[9] Ahmed G.A., Abdallah M.M., Abdelrahman M.A., Sharaf Eldin A.A. (2024). Effect of Pre-Procedural Statin/Ezetimibe Combination Therapy Versus Statin Monotherapy on Myocardial No-Reflow Following Percutaneous Coronary Intervention in Patients with Acute ST-Segment Elevation Myocardial Infarction: A Randomized Controlled Clinical Trial. QJM Int. J. Med..

[10] Zhong X., Luo L., Wu J., Li W., Liu X., Ye T., Li Z., Shi P. (2025). Adhesion-Assisted Antioxidant-Engineered Mesenchymal Stromal Cells for Enhanced Cardiac Repair in Myocardial Infarction. ACS Nano.

[11] Li J., Yao Y., Zhou J., Yang Z., Qiu C., Lu Y., Xie J., Liu J., Jiang T., Kou Y. (2024). Epicardial transplantation of antioxidant polyurethane scaffold based human amniotic epithelial stem cell patch for myocardial infarction treatment. Nat. Commun..

[12] Hua Y., He Z., Ni Y., Sun L., Wang R., Li Y., Li X., Jiang G. (2024). Silk fibroin and hydroxypropyl cellulose composite injectable hydrogel-containing extracellular vesicles for myocardial infarction repair. Biomed. Phys. Eng. Express.

[13] Zhu J., Du Y., Backman L.J., Chen J., Ouyang H., Zhang W. (2025). Cellular Interactions and Biological Effects of Silk Fibroin: Implications for Tissue Engineering and Regenerative Medicine. Small.

[14] Lin D., Li M., Wang L., Cheng J., Yang Y., Wang H., Ye J., Liu Y. (2024). Multifunctional Hydrogel Based on Silk Fibroin Promotes Tissue Repair and Regeneration. Adv. Funct. Mater..

[15] Vettori L., Tran H.A., Mahmodi H., Filipe E.C., Wyllie K., Liu Chung Ming C., Cox T.R., Tipper J., Kabakova I.V., Rnjak-Kovacina J. (2024). Silk fibroin increases the elasticity of alginate-gelatin hydrogels and regulates cardiac cell contractile function in cardiac bioinks. Biofabrication.

[16] Hassan S., Rezaei Z., Luna E., Yilmaz-Aykut D., Lee M.C., Perea A.M., Jamaiyar A., Bassous N., Hirano M., Tourk F.M. (2024). Injectable Self-Oxygenating Cardio-Protective and Tissue Adhesive Silk-Based Hydrogel for Alleviating Ischemia After Mi Injury. Small.

[17] Ni Y., Hua Y., He Z., Hu W., Chen Z., Wang D., Li X., Sun Y., Jiang G. (2024). Release of exosomes from injectable silk fibroin and alginate composite hydrogel for treatment of myocardial infarction. J. Biomater. Appl..

[18] Hu D., Li T., Bian H., Liu H., Wang P., Wang Y., Sun J. (2024). Silk films with distinct surface topography modulate plasma membrane curvature to polarize macrophages. Mater. Today Bio.

[19] Liu R., Zhao E., Wang Y., Zuo H., Li L., Xia Q., He H. (2025). Silk-engineered bioactive nanoparticles for targeted alleviation of acute inflammatory disease via macrophage reprogramming. J. Nanobiotechnol..

[20] Qian Z., Sun C., Li Q., Xie Y., Zhan L., Liu X., Wang G., Wei Y., Qiu J., Peng Q. (2024). Unravelling the antioxidant behaviour of self-assembly β-Sheet in silk fibroin. Redox Biol..

[21] Zhang R., Li Y., Ali U., Li Y., Zhang H. (2025). Unveiling novel antioxidant peptides from silk fibroin proteins: An integrated in silico and in vitro study. Food Chem..

[22] Wu S., Zhou X., Ai Y. (2023). Pro-angiogenic photo-crosslinked silk fibroin hydrogel: A potential candidate for repairing alveolar bone defects. J. Appl. Oral Sci..

[23] Watchararot T., Prasongchean W., Thongnuek P. (2021). Angiogenic property of silk fibroin scaffolds with adipose-derived stem cells on chick chorioallantoic membrane. R. Soc. Open Sci..

[24] Li Z., Tan G., Xie H., Lu S. (2024). The Application of Regenerated Silk Fibroin in Tissue Repair. Materials.

[25] Wang Y., Ye F., Wei X., Wang M., Xing Z., Liu H. (2025). Electrospun Silk Fibroin-Silk Sericin Scaffolds Induced Macrophage Polarization and Vascularization for Volumetric Muscle Loss Injury. J. Funct. Biomater..

[26] Gao Y., Wang Y., Zhang J., Zhang M., Dai C., Zhang Y., Zhang L., Bian L., Yang Y., Zhang K. (2024). Advancing neural regeneration via adaptable hydrogels: Enriched with Mg(2+) and silk fibroin to facilitate endogenous cell infiltration and macrophage polarization. Bioact. Mater..

[27] Manoharan C., Thomas D.S., Yashwant R.S., Roy G., Kunjupillai V., Mishra R.K., Nongthomba U., Gopalapillai R. (2025). Genetically engineered silk fibroin with antibacterial and antioxidant properties. 3 Biotech.

[28] Gavrilova N.A., Borzenok S.A., Revishchin A.V., Tishchenko O.E., Ostrovkiy D.S., Bobrova M.M., Safonova L.A., Efimov A.E., Agapova O.I., Agammedov M.B. (2021). The effect of biodegradable silk fibroin-based scaffolds containing glial cell line-derived neurotrophic factor (GDNF) on the corneal regeneration process. Int. J. Biol. Macromol..

[29] Aykac A., Karanlik B., Sehirli A.O. (2018). Protective effect of silk fibroin in burn injury in rat model. Gene.

[30] Park J.H., Cho K.I., Nam H., Choe N.-H., Suh J.-G. (2015). Anti-apoptotic effects of silk fibroin hydrolysate in RIN5F cell on high glucose condition. Anim. Cells Syst..

[31] Yang C., Yu R., Zhang Y., Wang Q., Huang D., Cheng Y., Zhu Y., Shen X., Shi Y., Zhao Y.Z. (2025). Curcumin-loaded bioadhesive silk fibroin microsphere improves islet transplantation by mitigating oxidative stress and inhibiting apoptosis. Mater. Today Bio.

[32] Song Y., Wang H., Yue F., Lv Q., Cai B., Dong N., Wang Z., Wang L. (2020). Silk-Based Biomaterials for Cardiac Tissue Engineering. Adv. healthc. Mater.

[33] Eliwa A., Abbas M.K.G., Al-Ejji M. (2025). Advancing cardiac patch viability and functionality: Innovations in scaffold design and cellular optimization. J. Mater. Sci. Mater. Med..

[34] Zahedi P., Hassani Besheli N., Farokhi M., Mottaghitalab F., Sohrabi A., Ghorbanian S.A. (2022). Silk Fibroin Nanoparticles Functionalized with Fibronectin for Release of Vascular Endothelial Growth Factor to Enhance Angiogenesis. J. Nat. Fibers.

[35] Bian Q., Chen J., Weng Y., Li S. (2022). Endothelialization strategy of implant materials surface: The newest research in recent 5 years. J. Appl. Biomater. Funct. Mater..

[36] Ma L., Dong W., Lai E., Wang J. (2024). Silk fibroin-based scaffolds for tissue engineering. Front. Bioeng. Biotechnol..

[37] Settembrini A., Buongiovanni G., Settembrini P., Alessandrino A., Freddi G., Vettor G., Martelli E. (2023). In-vivo evaluation of silk fibroin small-diameter vascular grafts: State of art of preclinical studies and animal models. Front. Surg..

[38] Jia J., Zhao X.A., Tao S.M., Wang J.W., Zhang R.L., Dai H.L., Zhang X.J., Han M.H., Yang B., Li Y. (2023). Icariin improves cardiac function and remodeling via the TGF-β1/Smad signaling pathway in rats following myocardial infarction. Eur. J. Med. Res..

[39] Feng Y., Zhao G., Xu M., Xing X., Yang L., Ma Y., Qi M., Zhang X., Gao D. (2021). rGO/Silk Fibroin-Modified Nanofibrous Patches Prevent Ventricular Remodeling via Yap/Taz-TGFβ1/Smads Signaling After Myocardial Infarction in Rats. Front. Cardiovasc. Med..

[40] Wang J., Wu Y., Wang Y., Shuai Y., Xu Z., Wan Q., Chen Y., Yang M. (2023). Graphene Oxide-Coated Patterned Silk Fibroin Films Promote Cell Adhesion and Induce Cardiomyogenic Differentiation of Human Mesenchymal Stem Cells. Biomolecules.

[41] Liu B., Zhang L., Guan X., Liu J., Shou W., Chen X., Li X., Cao D. (2025). Interpenetrating network hydrogel-loaded embryonic stem cell-derived endocardial cells improves cardiac function after myocardial infarction. J. Transl. Med..

[42] Yin Q., Zhu P., Liu W., Gao Z., Zhao L., Wang C., Li S., Zhu M., Zhang Q., Zhang X. (2023). A Conductive Bioengineered Cardiac Patch for Myocardial Infarction Treatment by Improving Tissue Electrical Integrity. Adv. healthc. Mater..

[43] Song Y., Zhang C., Zhang J., Sun N., Huang K., Li H., Wang Z., Huang K., Wang L. (2016). An injectable silk sericin hydrogel promotes cardiac functional recovery after ischemic myocardial infarction. Acta Biomater..

[44] Lo H.Y., Huang A.L., Lee P.C., Chung T.W., Wang S.S. (2018). Morphological transformation of hBMSC from 2D monolayer to 3D microtissue on low-crystallinity SF-PCL patch with promotion of cardiomyogenesis. J. Tissue Eng. Regen. Med..

[45] Mehrotra S., de Melo B.A.G., Miscuglio M., Kiaee K., Shin S.R., Mandal B.B. (2022). Mimicking Native Heart Tissue Physiology and Pathology in Silk Fibroin Constructs through a Perfusion-Based Dynamic Mechanical Stimulation Microdevice. Adv. healthc. Mater..

[46] Bhaarathy V., Venugopal J., Gandhimathi C., Ponpandian N., Mangalaraj D., Ramakrishna S. (2014). Biologically improved nanofibrous scaffolds for cardiac tissue engineering. Mater. Sci. Eng. C Mater. Biol. Appl..

[47] Nazari H., Heirani-Tabasi A., Hajiabbas M., Salimi Bani M., Nazari M., Pirhajati Mahabadi V., Rad I., Kehtari M., Ahmadi Tafti S.H., Soleimani M. (2020). Incorporation of SPION-casein core-shells into silk-fibroin nanofibers for cardiac tissue engineering. J. Cell. Biochem..

[48] Chi N.H., Yang M.C., Chung T.W., Chou N.K., Wang S.S. (2013). Cardiac repair using chitosan-hyaluronan/silk fibroin patches in a rat heart model with myocardial infarction. Carbohydr. Polym..

[49] Tsui J.H., Ostrovsky-Snider N.A., Yama D.M.P., Donohue J.D., Choi J.S., Chavanachat R., Larson J.D., Murphy A.R., Kim D.H. (2018). Conductive Silk-Polypyrrole Composite Scaffolds with Bioinspired Nanotopographic Cues for Cardiac Tissue Engineering. J. Mater. Chem. B.

[50] Wei X., Wang L., Duan C., Chen K., Li X., Guo X., Chen P., Liu H., Fan Y. (2023). Cardiac patches made of brown adipose-derived stem cell sheets and conductive electrospun nanofibers restore infarcted heart for ischemic myocardial infarction. Bioact. Mater..

[51] Liu J., Shen Y., Duan K., He X., Wang R., Chen Y., Li R., Sun J., Qiu X., Chen T. (2025). Novel biomimetic sandwich-structured electrospun cardiac patches with moderate adhesiveness and excellent electrical conductivity. J. Mech. Behav. Biomed. Mater..

[52] Gotoh Y., Yamazaki T., Ishizuka Y., Ise H. (2021). Interactions of N-acetyl-D-glucosamine-conjugated silk fibroin with lectins, cytoskeletal proteins and cardiomyocytes. Coll. Surf. B Biointerfaces.

[53] Liu W., Sun Y., Dong X., Yin Q., Zhu H., Li S., Zhou J., Wang C. (2020). Cell-derived extracellular matrix-coated silk fibroin scaffold for cardiogenesis of brown adipose stem cells through modulation of TGF-β pathway. Regen. Biomater..

[54] Cetin Y., Sahin M.G., Kok F.N. (2021). Application potential of three-dimensional silk fibroin scaffold using mesenchymal stem cells for cardiac regeneration. J. Biomater. Appl..

[55] Chi N.H., Yang M.C., Chung T.W., Chen J.Y., Chou N.K., Wang S.S. (2012). Cardiac repair achieved by bone marrow mesenchymal stem cells/silk fibroin/hyaluronic acid patches in a rat of myocardial infarction model. Biomaterials.

[56] Chen J., Zhan Y., Wang Y., Han D., Tao B., Luo Z., Ma S., Wang Q., Li X., Fan L. (2018). Chitosan/silk fibroin modified nanofibrous patches with mesenchymal stem cells prevent heart remodeling post-myocardial infarction in rats. Acta Biomater..

[57] Du M., Gu J., Wang J., Xue Y., Ma Y., Mo X., Xue S. (2019). Silk fibroin/poly(L-lactic acid-co-ε-caprolactone) electrospun nanofibrous scaffolds exert a protective effect following myocardial infarction. Exp. Ther. Med..

[58] Liang Y., Mitriashkin A., Lim T.T., Goh J.C. (2021). Conductive polypyrrole-encapsulated silk fibroin fibers for cardiac tissue engineering. Biomaterials.

[59] Tufan Y., Öztatlı H., Doganay D., Buyuksungur A., Cicek M.O., Döş İ., Berberoğlu Ç., Unalan H.E., Garipcan B., Ercan B. (2023). Multifunctional Silk Fibroin/Carbon Nanofiber Scaffolds for In Vitro Cardiomyogenic Differentiation of Induced Pluripotent Stem Cells and Energy Harvesting from Simulated Cardiac Motion. ACS Appl. Mater. Interfaces.

[60] Leite F.G., Marana J.F., de Sá L.F.T., Alves de Almeida T.F.R., do Carmo H.R.P., Chaud M.V., Grotto D., Silveira-Filho L.D.M. (2022). Effects of a collagen hyaluronic acid silk-fibroin patch with the electroconductive element polyaniline on left ventricular remodeling in an infarct heart model. J. Biomed. Mater. Res. B Appl. Biomater..

[61] Hu Y.F., Lee A.S., Chang S.L., Lin S.F., Weng C.H., Lo H.Y., Chou P.C., Tsai Y.N., Sung Y.L., Chen C.C. (2022). Biomaterial-induced conversion of quiescent cardiomyocytes into pacemaker cells in rats. Nat. Biomed. Eng..

[62] Kambe Y., Yamaoka T. (2019). Biodegradation of injectable silk fibroin hydrogel prevents negative left ventricular remodeling after myocardial infarction. Biomater. Sci..

[63] Ciocci M., Cacciotti I., Seliktar D., Melino S. (2018). Injectable silk fibroin hydrogels functionalized with microspheres as adult stem cells-carrier systems. Int. J. Biol. Macromol..

[64] Tapeh S.M.T., Baei M.S., Keshel S.H. (2021). Synthesis of thermogel modified with biomaterials as carrier for hUSSCs differentiation into cardiac cells: Physicomechanical and biological assessment. Mater. Sci. Eng. C Mater. Biol. Appl..

[65] Wu Y., Wang L., Guo B., Ma P.X. (2017). Interwoven Aligned Conductive Nanofiber Yarn/Hydrogel Composite Scaffolds for Engineered 3D Cardiac Anisotropy. ACS Nano.

[66] Song Z., Dong Z., Yu X., Li Y., Bai Y., Feng L., Qiao G., Wang X., Bi S. (2025). Bioengineered multifunctional hydrogel integrating oxygen sustention, oxidative stress alleviation and pro-angiogenic cues for regenerative MI therapy. Chem. Eng. J..

[67] Stoppel W.L., Gao A.E., Greaney A.M., Partlow B.P., Bretherton R.C., Kaplan D.L., Black L.D. (2016). Elastic, silk-cardiac extracellular matrix hydrogels exhibit time-dependent stiffening that modulates cardiac fibroblast response. J. Biomed. Mater. Res. A.

[68] Feng J., Wu Y., Chen W., Li J., Wang X., Chen Y., Yu Y., Shen Z., Zhang Y. (2020). Sustained release of bioactive IGF-1 from a silk fibroin microsphere-based injectable alginate hydrogel for the treatment of myocardial infarction. J. Mater. Chem. B.

[69] Asakura T. (2021). Structure of Silk I (Bombyx mori Silk Fibroin before Spinning)-Type II β-Turn, Not α-Helix. Molecules.

[70] R. Dan F., SH V., MS A., RK A., Kasoju N. (2023). A study on source dependent batch to batch variations in silk fibroin films for potential applications in corneal tissue engineering. Med. Comm. Biomater. Appl..

[71] Nicodemo D., Oliveira J.E., Sedano A.A., Marconcini J.M., Tonoli G.H.D. (2014). Impact of different silkworm dietary supplements on its silk performance. J. Mater. Sci..

[72] Yao X., Zou S., Fan S., Niu Q., Zhang Y. (2022). Bioinspired silk fibroin materials: From silk building blocks extraction and reconstruction to advanced biomedical applications. Mater. Today Bio.

[73] Huang L., Shi J., Zhou W., Zhang Q. (2023). Advances in Preparation and Properties of Regenerated Silk Fibroin. Int. J. Mol. Sci..

[74] Malashin I., Martysyuk D., Tynchenko V., Gantimurov A., Semikolenov A., Nelyub V., Borodulin A. (2024). Machine Learning-Based Process Optimization in Biopolymer Manufacturing: A Review. Polymers.

[75] El Seoud O.A., Kostag M., Possidonio S., Dignani M.T., Pires P.A.R., Lourenço M.C. (2022). Dissolution of Silk Fibroin in Mixtures of Ionic Liquids and Dimethyl Sulfoxide: On the Relative Importance of Temperature and Binary Solvent Composition. Polymers.

[76] Hu Y., Liu L., Yu J., Wang Z., Fan Y. (2020). Preparation of Natural Multicompatible Silk Nanofibers by Green Deep Eutectic Solvent Treatment. ACS Sustain. Chem. Eng..

[77] Shen T., Wang T., Cheng G., Huang L., Chen L., Wu D. (2018). Dissolution behavior of silk fibroin in a low concentration CaCl_2_-methanol solvent: From morphology to nanostructure. Int. J. Biol. Macromol..

[78] Juarez-Navarro K.J., Guarino V., Alvarez-Perez M.A. (2025). Converging Electrospinning and 3D-Printing Technologies: From Innovative Design for Tissue Engineering to Global Patent Trends and Technology Transfer. Fibers.

[79] dos Santos F.V., Siqueira R.L., de Morais Ramos L., Yoshioka S.A., Branciforti M.C., Correa D.S. (2024). Silk fibroin-derived electrospun materials for biomedical applications: A review. Int. J. Biol. Macromol..

[80] Yang C., Li S., Huang X., Chen X., Shan H., Chen X., Tao L., Zhang M. (2022). Silk Fibroin Hydrogels Could Be Therapeutic Biomaterials for Neurological Diseases. Oxid. Med. Cell. Longev..

[81] Zhang Y., Roohani I. (2025). Recent Advances in Silk Fibroin Derived from Bombyx mori for Regenerative Medicine. J. Funct. Biomater..

[82] Shen C., Wang J., Li G., Hao S., Wu Y., Song P., Han Y., Li M., Wang G., Xu K. (2024). Boosting cartilage repair with silk fibroin-DNA hydrogel-based cartilage organoid precursor. Bioact. Mater..

[83] Shen C., Zhou Z., Li R., Yang S., Zhou D., Zhou F., Geng Z., Su J. (2025). Silk fibroin-based hydrogels for cartilage organoids in osteoarthritis treatment. Theranostics.

[84] Mancini J.A., Compean A.L., Wagner D.T., Slocik J.M., Dennis P.B., Farajollahi S., Mirau P.A., Hoffmann A., Ruiz O.N., Gupta M.K. (2025). Enzymatic Halogenation of Silk Fibroin from Bombyx mori. ACS Omega.

[85] Bitar L., Jansing J., Isella B., Kopp A., Bortesi L. (2025). Recombinant production of silk fibroin in Nicotiana benthamiana using a modular library for transgene assembly. New Biotechnol..

[86] Fakhr M.J., Dezfouli M.R.M., Chaleshtori S.S. (2023). Animal Models and Methods of Myocardial Infarction Induction and the Role of Tissue Engineering in the Regeneration of Damaged Myocardium. Curr. Stem Cell Res. Ther..

[87] Zhang W., Chen L., Chen J., Wang L., Gui X., Ran J., Xu G., Zhao H., Zeng M., Ji J. (2017). Silk Fibroin Biomaterial Shows Safe and Effective Wound Healing in Animal Models and a Randomized Controlled Clinical Trial. Adv. healthc. Mater..

